# The “General practitioner learning stations”—development, implementation and optimization of an innovative format for sustainable teaching in general practice

**DOI:** 10.1186/s12909-021-03057-0

**Published:** 2021-12-16

**Authors:** Oxana Atmann, Marion Torge, Antonius Schneider

**Affiliations:** Technical University of Munich, TUM School of Medicine, Institute of General Practice and Health Services Research, Orleansstrasse 47, 81667 Munich, Germany

**Keywords:** Teaching general practice, Teaching, GP as a medical educator

## Abstract

**Background:**

Teaching general practice in a university setting is still challenging. In our department we have developed a teaching format with content from a previous lecture-style-teaching into an interactive small group format taught by frontline general practitioners (GPs). The “GP learning stations” introduce students to the skills and attributes of a GP working in primary care in a university setting. Our main objective was to understand whether the teaching format had proven itself sustainable in a university setting over eight years. Furthermore, we wanted to better understand the role of the GP as a medical educator.

**Methods:**

More than eight years of experience in organizational and staff expenses were collected and analyzed. In addition, the grade point average of the students’ evaluation was calculated and their free text answers were categorized and evaluated descriptively. During two teach-the-teacher seminars attending GPs were asked why they teach and if they feel equipped to teach the format.

**Results:**

The initially high organizational and staff expenses were significantly reduced. The recruitment of GPs, their didactic contribution, and their joint creation of content went smoothly throughout the whole period. A total of 495 students participated in the regular evaluation. The analysis yielded a grade point average of 1.9, on a scale from 1 = very good to 6 = insufficient. In the free text answers students praised the educators, the format and the practical relevance. The interactive transfer of the content, the didactic competence of the educators and the spatial environment were viewed critically. Reasons for GPs to teach were the joy to pass on knowledge and experience, and to make the work of GPs more attractive to students. Most GPs felt prepared to teach through their experience as a physician although some felt unprepared to teach through their lack of didactic knowledge.

**Conclusion:**

Despite reducing the costs of the format, a grade point average of 1.9 could be achieved in the long term. This supports the teaching concept of learning stations and its “mixture of discussion, scientific background and role play, combined with (…) experiences and exciting individual cases from (GPs) everyday life”, hopefully making general practice more attractive to the students.

## Background

For many medical students, becoming a general practitioner (GP) is still not a preferred career choice [1, 2]. In many countries this is a challenge in maintaining an adequate primary care system [3, 4]. However, the work of GPs and adequate primary care have an impact on hospital admissions, mortality, health outcomes and life expectancy [5–7]. Therefore, it is important that medical universities promote general practice as a career choice for students. Undergraduate medical education influences students career choice [8]. Factors known to have an influence are perception of the job, preferred working hours and work-life balance [9]. When considering the Bland-Meurer Model, the choice of specialty is essentially determined by the correspondence of personal career needs with students’ perception of specialty characteristics [10]. Moreover, exposure to charismatic role models and observing academic opportunities are also important drivers of career choices [11, 12].

In Germany, not all medical schools have a general practice department [13]. It has been shown that the interest of students in general practice is higher at universities with an established department [14]. Education in general practice for German medical students consists of three compulsory parts: [1] lectures at the university for all students with a total of around 15 h on different topics and days, [2] an individual two-week clerkship in an affiliated practice of the medical faculty, embedded in the current curriculum of the medical faculty and [3] an additional one-month clerkship in a general practice chosen by the students according to their interests, wherever they want.

It is also valuable to understand the emerging role of the practicing GP as a medical educator. Due to their numerous obligations in their practices, GPs are typically short of time and teaching at an academic institution or in the practice may not be a priority. Therefore, it is important to understand the relationship between the GP and the academic institution, and strike an appropriate balance between teaching time and didactic training of GPs in order to improve teaching skills.

Structured teaching formats can have a positive effect. It is unclear which teaching format can address this shortcoming and make a career as a general practitioner more attractive to medical students and strengthen the role of a GP as a medical educator. This article presents the experiences and insights of a structured teaching format “GP learning stations” which is used at our department to teach primary care to undergraduates in a university setting. We would also like to address whether the teaching concept as a whole has proven itself sustainable and suitable for students and general practitioners.

## Methods

### Conceptual format of the “GP learning stations”

**Fig. 1:**
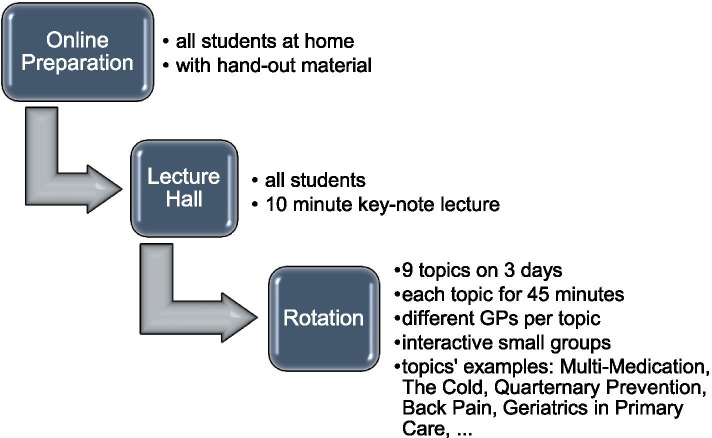
Basic structure of the “GP learning stations”

The “GP learning stations” are a compulsory format for interactive small groups of ten students in their 2^nd^ clinical year of study, with around 150 students each semester in various lecture halls of the medical university (see Fig. 1). On three Wednesday afternoons, students rotate within a time frame of 3.5 h through a total of nine topics, three per day. Topics are selected based on the expert consensus opinion of our GPs as a representative selection of general practice and updated as clinical guidelines change. Didactic elements are changed if this is deemed necessary to improve the interactive character of the learning stations. At the beginning of each day, students receive a 10-min key-note lecture together. Students are then divided into groups of ten and assigned to the learning station where they begin the rotation. After 45 min, the students rotate between the learning stations of the particular day. The learning stations are held by experienced, practicing general practitioners. Each day concludes with a take-home message and a question-and-answer session between the medical educators and students. Before the three-day format begins, students are asked to use online material provided to prepare the content, similar to the flipped classroom model. The objective of the format is to apply acquired knowledge and skills, the opportunity to meet large numbers of practicing GPs and to learn a professional attitude through practical and supervised interactions with symptoms and diseases.

**Fig. 2:**
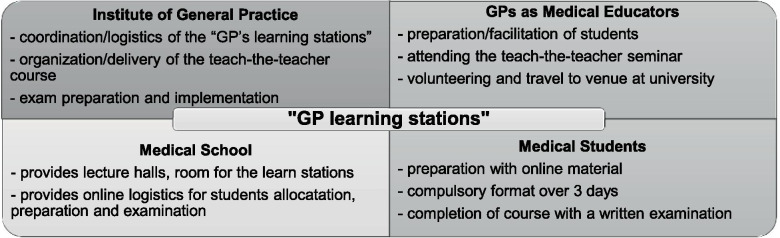
Stakeholders and their roles of the “GP learning stations”

Before the beginning of each semester, GPs can attend a five-hour teach-the-teacher seminar with a mixture of didactic training and medical content relevant to general practice in order to prepare for the “GP learning stations”. Four main stakeholders are involved in the “GP learning stations”: the institute of general practice, the GPs as medical educators, the medical school and the medical students. All stakeholders involved and their key work are shown in Fig. 2.

### Development

The development of the “GP learning stations” had two phases, an implementation phase (2011/12 to 2017), and an ongoing second optimization phase (2017/18 to 2018/19). In 2011, the prototype for teaching general practice in a clinical setting was developed and implemented by a group of practicing general practitioners under supervision of the Technical University of Munich Institute of didactics and then published to present the format to a wider audience [15]. Up until that point, general practice at our university consisted of lecture-style-teachings and a two-week clerkship in a general practice.

Recruitment of around 30 GPs from the state of Bavaria as medical educators proved to be unproblematic. The topics for the individual “GP learning stations” were determined by expert consensus between the general practitioners. Medical educators are provided with this pre-defined content and precise instructions for the small group interactions. General practitioners receive teaching material on each of the nine topics via an online platform and practical material at each learning station. The material contains a one-page basic information with learning objectives and a take-home message, as well as additional material for each topic. During the “GP learning stations”, GPs also receive practical material that they can use together with the students. For example, heavy gloves and cream-smeared sunglasses are part of the instant-aging practical material used at the geriatric learning station. With this disguise, students then have to remove tablets from a blister to understand a possible perspective of elderly people with poor eyesight and less precise hand feeling. The following table (Table 1) gives an overview of the learning stations and some of their numerous practical materials. Ideally, each learning station has a different GP, so that students have the opportunity to interact informally with as many GPs as possible and learn about the different attitudes and skillsets of GPs.

**Table 1:** Overview of highlights of the "GP learning stations"

**Highlights of the “GP learning stations”**	
**Main Topic**	**Examples of the work in small groups**
**Day 1 Acute and Chronic Illnesses in General Practice**	
Common cold and dyspnea	Dyspnea-self-awareness-exercise for the students who walk upstairs while breathing through a straw to experience dyspnea firsthand
Chest pain in general practice	Interactive work on the differences in the causes of chest pain
Diabetes in general practice	Hands-on examination of the feet with monofilament und the neurological tuning fork
**Day 2 Complex Consulting Occasions**	
Back pain in general practice	Case discussion on the treatment of back pain patient based on the National Care Guideline with a distinction between red, yellow, blue and black flags. Also, students practice practical physical exercises for non-specific back pain with mats and on transportable loungers
Quaternary prevention in general practice	Get to know the (rather unknown) term “Quaternary prevention” using four typical examples in general practice situations in order to raise awareness of prevention of unnecessary treatments and/or diagnostics or over-medication and to explore the basic medical principle of “primum non nocere”
Addiction in general practice or the “challenging” patient	Role play between a GP and an alcoholic or drug addicted patient; the GP helps the patient with empathy or withstands the pressure of the patient
**Day 3 The Elderly Patient**	
Multi-medication in general practice	The medication of a doctor's letter from a hospital is critically discussed and the medication is reduced in order to avoid possible interactions and to improve patient compliance
Geriatrics in general practice	Instant aging “light” experience with heavy gloves and cream-smeared sunglasses, with this disguise students have to remove tablets from a blister to understand a possible perspective of elderly people with poor eyesight and less precise hand feeling”
Palliative care in general practice	Interactive case discussion of a patient who comes from the clinic with a poor prognosis – how do general practitioners guarantee optimal care in the primary care sector?

During the implementation phase (2011/12 to 2017), the original format presented many challenges and was changed several times. The main problems were the extensive consumption of organizational resources, the lack of space to facilitate small groups for a large number of students, the allocation of all students to small groups, a small teaching and organization team at the institute and the appropriate selection of the content. For example, the large amount of practical material required a large number of student assistants just to bring the material from the institute to the lecture halls and set it up. Examples of resources are listed in Table 2. Due to the excessive consumption of organizational resources required for the “GP learning stations”, the format was structurally and logistically changed extensively during the implementation phase from 2011 to 2017. Starting with the optimization phase (2017/18 to 2018/19), the format evolved into a more structured format that was easier to implement with high quality and lower resource consumption.

**Table 2:** Examples of resources

Example of Resource	Starting Year	Implementation Phase	Optimization phase
Number of student assistants for preparation	3	3	1
Number of student-hours for preparation	195 h	195 h	65 h
Number of student assistants at each event	15	5	3
Number of student-hours at each event	180 h	90 h	54 h
Number of GPs needed for each day for the learning stations	30	27	27
Estimated catering costs and transport costs (depending on number of GPs and caterer)	3000 €	1100€	1000€
Estimated person-hours of GPs (non-paid)	810 h	364,5 h	364,5 h

The ongoing changes to the format are mainly made at the content level, due to regular changes in medical guidelines and regular updates in the medical field. Following feedback from students and GPs, changes are made to improve the interactive part. This keeps the format fluid, innovative and adaptable. Key elements of the development are shown in Table 3.

**Table 3:** The “GP Learning Stations”—from implementation to a standardized format

**Implementation phase** **(WS 2011/12 to SS 2017)**	**Optimization phase** **(WS 2017/18 to WS 2018/19)**
**Differences**	
•Different topics each semester •Variation of 35–45 min per session •Preparation for medical educators: Extensive material •Shortage of space to facilitate small groups	•Same topics each semester •Standardized time of 45 min per session •Preparation for medical educators: 1-page basic information with learning objectives and take-home message, plus additional material •More space: 3 lecture halls to facilitate 15 small groups
**Unchanged**	
•Access to online preparation material for students •Access to online preparation material for GPs •Online allocation of students to small groups •Medical educators attend the teach-the-teacher seminar each semester •Multiple Choice test at the end of the semester	

### The GP as a medical educator

General practitioners who facilitate the “GP learning stations” are physicians working in practices across the state of Bavaria/Germany. The participating GPs do not receive financial compensation for their facilitation. Hence, smart measures are needed to maintain the motivation to volunteer as a medical educator. Therefor we offer a teach-the-teacher seminar each semester prior to the beginning of the three-day “GP learning stations”, regular didactic training, the extensive Bavarian GP network and free access to the annual “Day of General Practice” organized by our institute. General Practitioners are recruited for the “GP learning stations” through the institute’s GP network. In order to improve the didactic competence of our medical educators during the teach-the-teacher seminar, didactic contents such as learning theory, use of media and practical material, presentation technology and giving feedback are conveyed. The didactic part of the teach-the-teacher seminars are based on the didactic content recommended by the Society of Medical Education (GMS) in Germany [16]. The teach-the-teacher seminar is based on a mix of student feedback, GP learning needs, recommended didactic skills and ongoing changes of medical guidelines. To better understand our cohort of educators, we asked GPs attending our biannual teach-the-teacher seminar why they teach in general practice and whether they feel prepared to teach general practice.

Both the teach-the-teacher seminar and the three-day “GP learning stations” take place on a Wednesday afternoon, as most practices in the primary care sector in Germany are closed on Wednesday afternoons to allow time for professional development and administrative tasks.

### Evaluation

At the Technical University of Munich medical school students regularly and anonymously evaluate each teaching unit. Evaluation takes place by means of giving a school grade (1 = very good to 6 = insufficient) and optional free text comments online through the medical school’s evaluation platform. The data for this work were obtained retrospectively from the regular evaluation. The grade point average of students’ evaluation was calculated for the optimization phase and their free text answers were categorized by main topics and evaluated descriptively. We have also evaluated the departments’ experiences over eight years with regard to organizational and staff resources for the “GP learning station”. In addition, once in September 2018 and once in September 2019, we asked all GPs (2018: 19 GPs, 2019: 17 GPs) attending our biannual teach-the-teacher seminar, to complete a brief questionnaire. We assessed the GPs’ age, distance travelled to the medical school, their educational experience, and reasons for teaching general practice, and if they felt prepared to teach. The data provided by the GPs were descriptively categorized according to main topics. One rater categorized the free-text answers and a second rater did the same. Then the categories were compared and in the event of discrepancies categories were further discussed and then determined in an expert group. Analysis was carried out with SPSS 24.0. Free text answers were summarized in categories and then counted according to frequencies.

## Results

### “GP learning stations”

Organizational and staff expenses were significantly reduced. From 20 changing topics in two semesters, we have reduced the format to 9 unvarying topics each semester. Instead of initially 15 medical student assistants who helped set up the “GP learning stations” for each event, only three are now required. Recruitment of GPs, didactic training and voluntary participation were consistent throughout the semesters. A total of 495 students participated in the optimization phase from 2017/18 to 2018/19. Analysis yielded a grade point average of 1.99 (Table 4) which is very similar to the grade average of 2.1 from the evaluation of the early phase in 2012/13 [17].

**Table 4:** Average grade given by students from 2017/18 to 2018/19

**Semester**	**Students/ Semester**	**Average** **grade**	**Total possible number of evaluations for 3 teaching days**	**Number of evaluations (free text answers)**	**Number of evaluations (grade)**
2017/18	162	1,92	486	51 (11%)	311 (64%)
2018	162	1,94	486	47 (10%)	315 (65%)
2018/19	171	2,08	513	54 (11%)	354 (69%)
total	495	1,99	1485	152 (10%)	980 (66%)

In the free text answers charismatic lecturers, the format and the practical relevance was praised. Content, didactic competence and spatial setting were mentioned critically. Some students criticized the more holistic and longer patients’ stories of GPs as “fairy tale stories” (see Table 5). Other students found it enriching to be able to interview different GPs in a small group setting. This could also be used as a concept in other departments.

**Table 5:** Categorized free text answers from students

**Categories of free text answers** (Total of 152 student answers, number/percent)	**Selection of common answers** **(Several categories per evaluation/student possible)**
Praise: Format (77/51%)	Good concept (28/18%), Content and topics interesting (18/12%), Small group interaction (10/5%), Fun (6/4%)
Praise: Great Lecturer (54/36%)	Nice and friendly (16/11%), Committed (9/6%), Personal contact (6/4%)
Praise: Practical (7/5%)	Learned a lot (2/1%)
Praise: Student friendly (3/2%)	
Criticism: Competence as a lecturer (52/34%)	Lack of preparation or unstructured (15/10%), bad time management (10/7%), unmotivated or ambiguous (8/5%), Does not appear didactic competent (5/3%), same lecturers during the 3 day event (5/3%)
Criticism: Content (45/30%)	Little knowledge gain (12/8%), redundant topics and basic (12/8%), “fairytale story time” (9/6%), promotional event (3/2%)
Criticism: Different lecturers (20/13%)	Varying quality between lecturers (8/5%), professional competence variable (5/3%), fluctuating cooperation between professionals (3/2%)
Criticism: Setting (19/13%)	Lecture hall not suitable (8/5%), too loud in the lecture hall (4/3%)
Notable quotes: Praise	- “Listen to the opinion or the way of working from nine different GPs” - “Extremely interesting to listen to this [content] again from practical experience and not to learn from slides or books. All in all a successful lecture” - “Impressive and very inspiring, could provide technical info and answer questions about the life and work of a family doctor to get an impressions of this specialization” - “Refreshing to have so many doctors as lecturers” - “I was convinced by both the GPs’ human and professional competence. I think even more now that this subject is the most demanding, because you have to have the widest knowledge and good diagnosing skills” - “She had the perfect mix of discussion, scientific background and role play, combined with her own experience and exciting individual cases from [GP] everyday life” - “The event is so practical,… you can directly ask GPs questions that you would rather not ask in a large group”
Notable quotes: Criticism	- “For effective teaching a certain preparation is also needed by experienced physicians” - “ Good will alone does not make a good course”

### The GP as a medical educator

A total number of 35 GPs completed the questionnaire, of which 37% were female, mean age was 55 years. The journey to the venue was approximately 45 km on average (Table 6). The most important reasons given for teaching general practice were the joy to pass on knowledge and experience to the students, to contribute to making general practice more attractive to students, to deal more often with medical guidelines and innovations and the exchange with colleagues (Table 6). GPs felt prepared to teach because of their long experience as a physician, their years of experience in teaching students and their didactic training. Some felt not well prepared to teach because they lacked didactic knowledge and due to the rapidly changing knowledge in medicine (Table 7).

**Table 6:** Characteristics of participating GPs

**Variables** (number/percent)	**Total**
Number of participants	35 (100%)
Gender (Female)	13 (37%)
Mean Age (years)	55 ± 8
Journey to venue (km)	45 ± 11
Why do you teach in General Practice?	
-Joy of teaching	28 (80%)
-Pass on knowledge and experience to the students	22 (63%)
-To contribute to making General Practice more attractive to students	20 (57%)
-Clinical professional development	16 (46%)
-Exchange with colleagues	15 (43%)
-Promotion of young doctors	10 (29%)
-Because good teaching is important	7 (20%)
-Because the “GP learning stations” are a good concept	2 (6%)
-Contact with the academic body of medicine	2 (6%)
-Because I can	1 (3%)
-Proximity to the students	1 (3%)
-To present the broad spectrum of General Practice	1 (3%)
-Because I like to do something different to just working in the practice	1 (3%)
-Because I appreciate the holistic view of the subject and the patient	1 (3%)

**Table 7:** Categorized free text answers from GPs

**Categories of free text answers** (Total of 35 GP answers, number/percent)
I feel prepared for teaching in General Practice
-Through my experience as a physician (17/ 49%)
-Because I have many years of experience in teaching students (12/34%)
-Through my didactic training (11/31%)
-Because I enjoy teaching (2/6%)
-Because I am well prepared through the trainings and material provided (2/6%)
-Because I feel motivated (2/6%)
I don’t feel prepared to teach in General Practice
-Because I feel I do not have enough didactic knowledge (4/11%)
-Because knowledge in medicine is rapidly changing (2/6%)
-Lack of time (1/3%)

## Discussion

Transferring medical knowledge from a primary care setting to students in a university hospital setting poses a number of challenges for departments of General Practice. One factor is that General Practice is still a new department for most medical universities in Germany and due to their small teams, or their non-existence at some medical faculties in Germany, GPs in academic medicine make up only a small proportion of the clinical academic setting [13]. Similar challenges are even seen in the UK with its strong primary care departments. For example, in the UK 6.5% of all clinical academics are GPs, Pereira Gray calling this a public failure by UK medical schools [18]. To date, no exact figures are available in Germany [13]. This could give medical students introduced to the medical world at university teaching hospitals the impression that general practice is not important. This contradicts the findings that continuity of care is important for patients and is associated with lower mortality rates, greater adherence to medical advice and decreased use of hospital services [19, 20]. Continuity of care is generally considered the task of general practitioners in the primary care workforce [21]. To sustain continuity of care and to prevent a crisis in the primary care workforce [22], medical schools should promote primary care at all stages of the medical school. Early, ongoing and authentic exposure promotes general practice [23].

Our format offers the opportunity to expose medical students at the beginning of their career to authentic role models, a large number of various GPs and topics specific to primary care. From the perspective of the students, the format has proven itself, as grades and comments show high acceptance. The format also worked well from the lecturer’s point of view, as we have been able to retain a large number of motivated educators over the years. This strengthens the role of GPs as medical educators. Hence, their motivation and their needs play an important role in sustaining such concepts and need to be further assessed [24]. Based on our experience and the responses we have received, we believe that GPs are interested in coming to medical school and teaching the students not only in their practice but also in the clinical setting where students spend a lot of time studying. We also assume that regular contact with their colleagues through a didactic event increases the attraction and pulls the GPs out of their practices in order to enjoy the long-term relationship with the academic institution. Of course, these assumptions need further evaluation.

Our brief survey during the teach-the-teacher seminar participants showed that the most important factors were the joy of teaching and the passing on of knowledge and experience to the students. Similar motivational factors have been found by Thomson et al. [25]. They encourage teaching organizations to give more credit to lecturers, emphasize the joy and pride in teaching, and to increase engagement with GPs. Furthermore, the general supportive environment could help foster and maintain a learning culture within workplace-based learning outside the hospital setting, for example for medical educators in practices.

Students praised the engagement to charismatic role models, which is a known factor in career choices, and criticized some didactic deficits of the lecturers. They pointed out that working as a GP does not necessarily qualify as a medical educator. The more holistic and lengthy patient stories of general practitioners have also been critically viewed as “fairy tale stories”. We can only hypothesize that the comments come from perceiving the difference in narrative between GPs and clinicians. This has to be quite a contrast to what the students are familiar with. Unlike in clinical settings, general practitioners often know their patients from cradle to grave, care for them multiple times and often know the family background.

However, GPs felt equipped to teach due to their longstanding practice. Few stated that they did not feel they had sufficient didactic knowledge. We try to remedy this deficit through our biannual teach-the-teacher seminar, as we know that a lack of time and the dual role of GPs as practicing doctors and medical educators place an additional burden on them. During these trainings, we emphasize the role change from knowledge mediator to an interactive learning companion, in which the students themselves develop essential content based on the tasks and materials. We believe that other ways of teaching GPs didactic skills should be further explored. We also see informal aspects of the “GP learning stations”, such as the binding of non-academic GPs to the academic body. Historically, most general practitioners in Germany are not affiliated with academic institutions. This may be because in the German health care system, a primary care practice acts like a small business in the hands of the GP or a group of GPs. In our experience, many general practitioners want to share their knowledge, skills and experience as a GP with the next generation of medical students [26]. Our “GP learning stations” in a clinical setting offer this opportunity with a low threshold. Furthermore, this connection can contribute to translate experience and knowledge through a constant exchange from the practices to the medical university and vice versa. For example, a research paper on colonoscopy in primary care was recently published through the binding of non-academic GPs to our department [27]. Further research into this relationship could help understand and strengthen the mutual benefits of this complex relation.

### Strengths and limitations

This paper not only presents the learnings of our department over a longer period of time, but also provides a format for introducing general practice to students in a clinical setting and influencing their perception of the specialty. Even though the format has its limitations due to its regional nature, it could serve as a model for “GP learning stations” at other universities. Unfortunately, due to the setting of the regular evaluation system of the medical school, students’ evaluation was not very extensive and limited to the grade as well as optional free text answers. In addition, the evaluation of GPs as medical educators was limited as the number of GPs participating was small and the participants very engaged. Since the article presents the learnings of the format in our context, its application in other contexts may be limited.

## Conclusion

The “GP learning stations” have proven themselves over time as a teaching format for general practice in a clinical setting and are improved by the ongoing evaluation of the students and medical educators. It may represent a structured teaching format that has the potential to enhance students career choice to become a GP. The survey of GPs has provided some insight into the motivating factors for teaching and potential obstacles. Despite the overall organizational and staff reduction within the format, a grade point average of 1.9 was achieved in the long term. This strengthens the “GP learning stations” as a teaching format in primary care and its “mixture of discussion, scientific background and role play, combined with (…) GPs experience and exciting individual cases from [GP] everyday life".

## Data Availability

The datasets generated and/or analysed during the presentation of the teaching format are not publicly available due to restrictions, but are available from the corresponding author on reasonable request and with permission of the Technical University of Munich, TUM School of Medicine.

## Abbreviations

GP: General Practitioner
MC: Multiple Choice
